# Trends in antenatal depression and suicidal ideation diagnoses among commercially insured childbearing individuals in the United States, 2008–2018

**DOI:** 10.1016/j.jad.2022.09.120

**Published:** 2022-09-27

**Authors:** K.M. Tabb, V.K. Dalton, A. Tilea, G.E. Kolenic, L.K. Admon, S.V. Hall, X. Zhang, K.K. Ryckman, K. Zivin

**Affiliations:** a University of Illinois at Urbana-Champaign, School of Social Work, Urbana, IL, United States of America; b University of Michigan, Department of Obstetrics and Gynecology, Ann Arbor, MI, United States of America; c University of Michigan, Department of Biostatistics, Ann Arbor, MI, United States of America; d University of Michigan, Department of Learning Health Sciences, Ann Arbor, MI, United States of America; e University of Iowa, College of Public Health, Iowa City, IA, United States of America; f Indiana University, School of Public Health, Bloomington, IN, United States of America; g Center for Clinical Management Research, VA Ann Arbor Healthcare System, Ann Arbor, MI, United States of America.

**Keywords:** Pregnancy, Depression, Suicidal ideation, Trends, Mental health

## Abstract

**Introduction:**

Antenatal depression and suicidal ideation represent serious pregnancy-related complications, yet comprehensive estimates of the prevalence and predictors of these diagnoses among birthing people remain unclear.

**Objective:**

This study aimed to characterize trends in the prevalence of depression and suicidal ideation diagnoses identified among pregnant individuals prior to giving birth.

**Methods:**

This study included 536,647 individuals aged 15–44 years continuously enrolled in a single commercial health insurance plan for one year before childbirth from 2008 to 2018. The primary outcomes included depression or suicidal ideation based on identification of the relevant ICD-9 and ICD-10 diagnosis codes during pregnancy.

**Results:**

Rates (95 % CIs) of depression increased by 39 % from 540 (520–560) per 10,000 individuals in 2008 to 750 (730–770) per 10,000 individuals in 2018. Suicidal ideation increased by 100 % from 15 (12–18) per 10,000 individuals in 2008 to 44 (39–50) per 10,000 individuals in 2018. Black birthing people experiencing the sharpest proportional increases.

**Conclusions:**

The prevalence of depression and suicidal ideation occurring during pregnancy substantially increased over a ten-year period. Further, suicidal ideation diagnosis increased the most for among Black birthing people compared to all groups, resulting in a need for future studies in this area to determine the reasons for an increase in diagnosis and any change in resulting treatment of follow up.

## 1. Introduction

Maternal mortality and morbidity steadily increased from 1990 through 2015 in the United States and up to two-thirds of cases may have been preventable if addressed earlier (Davis et al., 2019; Kassebaum et al., 2014). Depression and suicidal ideation are major risk factors for mortality or morbidity and represent serious complications during pregnancy (Cohen et al., 2004). Untreated depression during the perinatal period has profound effects on birthing people and their offspring (Ogunyemi et al., 2018; Simonovich et al., 2021). Past studies found racial differences in antenatal depressive symptoms and suicidal ideation where minority (Black and Asian) adults experience higher rates of depression symptoms (Gavin et al., 2011a; Gavin et al., 2011b). There is a discrepancy between rates of depressive symptoms and diagnosis rates in non-gravid populations, where racial minority adults have higher depressive symptoms, but are less likely to have a resulting diagnosis (Simpson et al., 2007). Past studies found that racial minority adults are more likely to have a substance use disorder diagnosis but less likely to receive a mood disorder diagnosis even when symptoms are present (Boyd et al., 2011; Delphin-Rittmon et al., 2015; Simpson et al., 2007).

To date, few studies have included diverse pregnant samples to document depression diagnoses during pregnancy. Previous studies on antenatal depression and suicidal ideation focused on regional samples, often limited to a single time point (Alhusen et al., 2015; Baer et al., 2016). This study aimed to characterize trends in depression and suicidal ideation diagnoses among racially and ethnically diverse pregnant individuals prior to giving birth over a ten-year period.

## 2. Methods

This study examined trends in depression and suicidal ideation diagnoses identified in the year before birth among individuals aged 15–44 using the Optum^™^ Clinformatics^™^ Data Mart (CDM). CDM is a statistically deidentified database of administrative medical claims for members across all 50 US states derived from a large claims data warehouse. We identified hospital deliveries from 2008 to 2018 and restricted the sample to those with continuous enrollment in a single employer-based health plan for at least 1 year before a live birth. We identified individuals with depression and suicidal ideation during pregnancy using standardized *International Classification of Disease-9th and 10th Revision- Clinical Modification* diagnosis and procedure codes present at least once in either inpatient or outpatient claims (CCS). We described demographic and clinical characteristics including age, insurance type, other mental health conditions, and substance use disorder for all individuals and for those with depression or suicidal ideation during pregnancy. Optum data contained a race/ethnicity classification including Black, White, Asian, Hispanic, and Other; outcome variation by this classification represents the primary predictor of interest. The data source includes race/ethnicity identified using a combination of self-report, public records, and demographic derivations based on a third-party algorithm (E-Tech). The University of Michigan Institutional Review Board approved this study.

## 3. Results

**Fig. 1.**
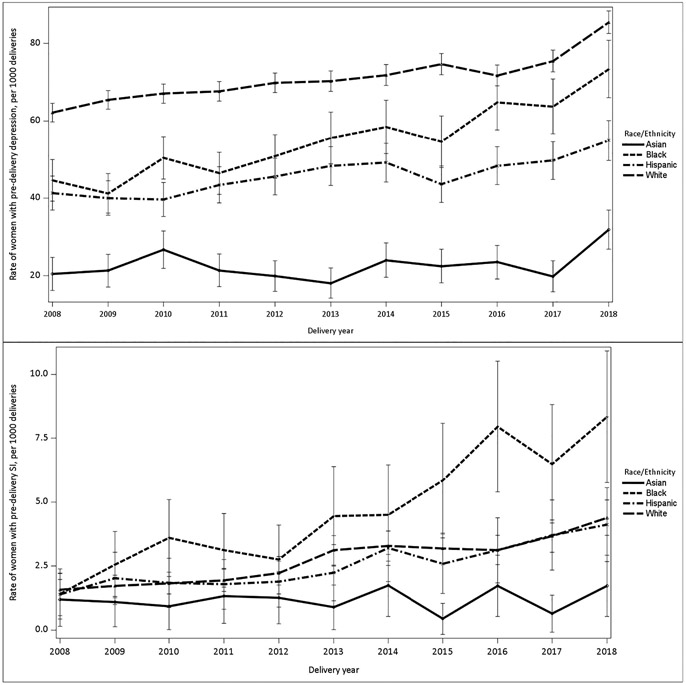
Trends in antenatal depression and antenatal suicidal ideation by race among commercially insured childbearing individuals, 2008–2018 (N = 536,647).

Of the 536,647 commercially insured childbearing individuals that met study criteria between 2008 and 2018, 36,766 (6.8 %) had a diagnosis for either depression or suicidal ideation during pregnancy over the study period. Rates (95 % CIs) of depression increased by 39 % from 540 (520–560) per 10,000 individuals in 2008 to 750 (730–770) per 10,000 individuals in 2018. Suicidal ideation increased by 193 % from 15 (12–18) per 10,000 individuals in 2008 to 44 (39–50) per 10,000 individuals in 2018 (Fig. 1.). Across all racial and ethnic categories, both antenatal depression and suicidal ideation increased over a ten-year period. Black birthing people experienced the sharpest proportional increases. Depression rates among Black birthing people rose by 66 % from 440 (390–490) per 10,000 individuals in 2008 to 730 (660–800) per 10,000 individuals in 2018. Suicidal ideation rates among Black birthing people rose by 700 % from 10 (4–24) per 10,000 individuals in 2008 to 80 (50–100) per 10,000 individuals in 2018 (Table 1).

## 4. Discussion

Depression and suicidal ideation diagnoses during pregnancy increased significantly between 2008 and 2018 in a sample of 536,647 commercially insured birthing people. The results provide an estimate of diagnosis rates of depression and suicidal ideation during pregnancy. Most studies on mental health during pregnancy limit describing the prevalence of depression symptomology to a single time point. This study found that an upward trend in depression diagnosis and suicidal ideation diagnosis, similar to trends suicidal ideation increasing among female adolescents and Black youth (Lindsey et al., 2019; Pontes et al., 2020) This study also describes trends over time and the differences in depression diagnosis and suicidal ideation diagnosis by racial and ethnic categories. The rate of depression diagnosis during pregnancy increased disproportionately across racial and ethnic groups, and Black birthing people experienced the sharpest increase. Suicidal ideation diagnosis increased the most for among Black birthing people compared to all groups, resulting in a need for future studies to determine the reasons behind this increase and any change in the resulting treatment of follow up.

A diagnosis can lead to treatment and mental health services, but many barriers, such as culturally responsive communication from providers, persist (Atdjian and Vega, 2005). Past studies documented the way minority adults communicate mental health problems with White providers and the limitations of White providers identifying mental health needs (Bailey et al., 2019). In a recent qualitative report, Black women were more likely to speak with their providers about thoughts of self-harm and suicidal ideation than depression because they perceived providers would only listen to them about serious topics of harm or imminent risks (Hsieh et al., 2021). The risk for reporting suicidal ideation can vary from the first pregnancy and across subsequent pregnancies (Kim et al., 2016). Kim and colleagues found that depression and suicidal ideation during a first pregnancy as strongly associated with depression and suicidal ideation during the postpartum period and recurrence in following pregnancies. By reporting the trends in diagnosis over time, this study presents a story of the overall increase of antenatal depression and suicidal ideation diagnoses.

Recognizing depression in birthing people during pregnancy represents an early, but necessary, step in the perinatal mental health treatment pathway (Byatt et al., 2015) to support individuals in the postpartum period and beyond. An increase in diagnoses could reflect a growing awareness of mental health conditions during pregnancy and its consequences. In addition, the overall increase might result from swift moving policy reform to integrate mental health into primary care settings.

This study has multiple strengths and some limitations. The study presented findings from a privately insured sample and does not provide estimates for rates of depression for publically insured individuals. More than half of births in the United States are covered under public insurance options and thus not including publically insured individuals is a limitation of this study. This study focused on depression and suicidal ideation diagnosis during pregnancy, whereas other studies found that the full spectrum of mental health problems such as bipolar disorder or trauma that can lead to adverse perinatal outcomes (Baer et al., 2016). Despite these limitations, an estimate of the trends in diagnoses over time is important for clinical decision making and evaluation of policy making to determine if diagnoses rates are increasing.

## 5. Conclusion

This study found increasing trends in antenatal depression and antenatal suicidal ideation diagnoses during pregnancy and the greatest increase exists for Black birthing people. Given recent societal events such as COVID-19 and the public death of George Floyd, these timely findings have clinical relevance. This study highlights the need to pay closer attention to minoritized birthing people, particularly Black women. Identifying patterns and trends in mental health conditions during pregnancy provides critical evidence to support early prevention and interventions in the postpartum period. Several studies find that suicidal ideation during the perinatal period has increased over time (Admon et al., 2021) and can be succesfully identified through depression screening practices (Kim et al., 2015; Kim et al., 2014). Diagnosis is during pregnancy important and remains essential to identify those in need of mental health treatment during pregnancy and possibly extending to the postpartum period.

## Figures and Tables

**Table 1:** Demographic characteristics of study sample (N = 536,647), overall and by antenatal depression and suicidal ideation, 2008–2018.

Category	All individuals 2008		All individuals 2018		Antenatal depression 2008		Antenatal depression 2018		Antenatal suicidal ideation 2008		Antenatal suicidal ideation 2018	
						
	N	% (95 % CI)	n	% (95 % CI)	n	% (95 % CI)	n	% (95 % CI)	n	% (95 % CI)	n	% (95 % CI)
*Age*												
≤18	1096	1.72% [0.95,2.50]	321	0.59% [−0.25,1.43]	80	7.30 % [5.76,8.84]	64	19.94 % [15.54,24.33]	16	1.46% [0.75,2.17]	23	7.17% [4.33,10.00]
19–26	9903	15.58 % [14.87,16.29]	8113	14.91 % [14.13,15.68]	508	5.13 % [4.70,5.56]	816	10.06 % [9.40,10.71]	32	0.32 % [0.21,0.43]	116	1.43% [1.17,1.69]
27–34	34,200	53.81 % [53.28,54.34]	29,231	53.71 % [53.14,54.28]	1674	4.89 % [4.67,5.12]	1856	6.35 % [6.07,6.63]	31	0.09 % [0.06,0.12]	59	0.20% [0.15,0.25]
35–39	14,470	22.77 % [22.08,23.45]	13,595	24.98 % [24.25,25.71]	863	5.96 % [5.58,6.35]	1011	7.44 % [7.00,7.88]	12	0.08 % [0.04,0.13]	32	0.24% [0.15,0.32]
≥ 40	3891	6.12% [5.37,6.88]	3161	5.81 % [4.99,6.62]	318	8.17% [7.31,9.03]	341	10.79 % [9.71,11.87]	7	0.18 % [0.05,0.31]	12	0.38 % [0.17,0.59]
*Race*												
Asian	4215	6.63 % [5.88,7.38]	4644	8.53 % [7.73,9.34]	86	2.04 % [1.61,2.47]	148	3.19% [2.68,3.69]	5	0.12% [0.01,0.22]	8	0.17% [0.05,0.29]
Black	5679	8.93 % [8.19,9.68]	4797	8.81 % [8.01,9.62]	253	4.46 % [3.92,4.99]	352	7.34 % [6.60,8.08]	8	0.14 % [0.04,0.24]	40	0.83 % [0.58,1.09]
Hispanic	7934	12.48% [11.76,13.21]	7524	13.83 % [13.05,14.61]	328	4.13 % [3.70,4.57]	413	5.49 % [4.97,6.00]	11	0.14 % [0.06,0.22]	31	0.41 % [0.27,0.56]
Unknown/missing	6940	10.92 % [10.19,11.65]	2603	4.78% [3.96,5.60]	366	5.27% [4.75,5.80]	197	7.57 % [6.55,8.58]	13	0.19 % [0.09,0.29]	10	0.38 % [0.15,0.62]
White	38,792	61.03 % [60.55,61.52]	34,853	64.04 % [63.54,64.55]	2410	6.21 % [5.97,6.45]	2978	8.54 % [8.25,8.84]	61	0.16 % [0.12,0.20]	153	0.44% [0.37,0.51]
*Income*												
≤400 % FPL	329	0.52 % [−0.26,1.29]	473	0.87% [0.03,1.71]	21	6.38 % [3.73,9.04]	37	7.82 % [5.39,10.25]			8	1.69% [0.53,2.86]
>400 % FPL	48,706	76.63 % [76.25,77.01]	41,961	77.10 % [76.70,77.51]	2659	5.46 % [5.26,5.66]	3209	7.65 % [7.39,7.90]	64	0.13 % [0.10,0.16]	167	0.40% [0.34,0.46]
*Region*												
Great Lakes/Northern Plains	15,110	23.77 % [23.09,24.45]	14,846	27.28 % [26.56,28.00]	972	6.43 % [6.04,6.82]	1391	9.37 % [8.90,9.84]	25	0.17 % [0.10,0.23]	92	0.62% [0.49,0.75]
Mountain	5745	9.04 % [8.30,9.78]	5695	10.46 % [9.67,11.26]	366	6.37 % [5.74,7.00]	491	8.62 % [7.89,9.35]	10	0.17 % [0.07,0.28]	25	0.44% [0.27,0.61]
Northeast	6441	10.13 % [9.40,10.87]	5375	9.88% [9.08,10.67]	391	6.07 % [5.49,6.65]	356	6.62 % [5.96,7.29]	8	0.12% [0.04,0.21]	10	0.19% [0.07,0.30]
Pacific	7100	11.17% [10.44,11.90]	6172	11.34 % [10.55,12.13]	244	3.44 % [3.01,3.86]	384	6.22 % [5.62,6.82]	5	0.07 % [0.01,0.13]	15	0.24% [0.12,0.37]
Southeast	29,107	45.79 % [45.22,46.37]	22,110	40.63 % [39.98,41.28]	1468	5.04 % [4.79,5.29]	1463	6.62 % [6.29,6.94]	50	0.17 % [0.12,0.22]	99	0.45% [0.36,0.54]
Unknown	57	0.09 % [−0.69,0.87]	223	0.41 % [−0.43,1.25]	2	3.51 % [−1.42,8.43]	3	1.35% [−0.18,2.87]			1	0.45% [−0.44,1.33]
*Insurance*												
POS	41,705	65.62 % [65.16,66.07]	40,318	74.09 % [73.66,74.51]	2260	5.42 % [5.20,5.64]	3002	7.45 % [7.19,7.70]	67	0.16 % [0.12,0.20]	159	0.39 % [0.33,0.46]
EPO or HMO	19,648	30.91 % [30.27,31.56]	12,603	23.16 % [22.42,23.89]	1009	5.14% [4.83,5.44]	887	7.04 % [6.59,7.48]	25	0.13 % [0.08,0.18]	53	0.42% [0.31,0.53]
PPO	2038	3.21 % [2.44,3.97]	772	1.42% [0.58,2.25]	148	7.26% [6.13,8.39]	82	10.62 % [8.44,12.80]	6	0.29 % [0.06,0.53]	12	1.55% [0.68,2.43]
Indemnity/other	169	0.27 % [−0.51,1.04]	728	1.34% [0.50,2.17]	26	15.38 % [9.89,20.88]	117	16.07 % [13.40,18.75]			18	2.47% [1.34,3.60]
*Other mental health conditions*												
No	59,377	93.42 % [93.22,93.62]	44,996	82.68 % [82.33,83.03]	1757	2.96 % [2.82,3.10]	1050	2.33 % [2.19,2.47]				
Yes	4183	6.58 % [5.83,7.33]	9425	17.32 % [16.55,18.08]	1686	40.31 % [38.82,41.79]	3038	32.23 % [31.29,33.18]				
*Substance-related disorder*												
No	60,882	95.79 % [95.63,95.95]	48,240	88.64 % [88.36,88.93]	2979	4.89 % [4.72,5.06]	2886	5.98 % [5.77,6.19]	54	0.09 % [0.07,0.11]	82	0.17% [0.13,0.21]
Yes	2678	4.21 % [3.45,4.97]	6181	11.36 % [10.57,12.15]	464	17.33 % [15.89,18.76]	1202	19.45 % [18.46,20.43]	44	1.64% [1.16,2.12]	160	2.59% [2.19,2.98]

FPL = federal poverty level; POS = point of service; EPO = exclusive provider organization; HMO = health maintenance organization; PPO = preferred provider organization.
